# Enhancing Clinical Decision Making by Predicting Readmission Risk in Patients With Heart Failure Using Machine Learning: Predictive Model Development Study

**DOI:** 10.2196/58812

**Published:** 2024-12-31

**Authors:** Xiangkui Jiang, Bingquan Wang

**Affiliations:** 1 School of Automation, Xi’an University of Posts and Telecommunications, No. 563 Chang'an South Road, Yanta District, Xi’an, Shaanxi, 710121, China, 86 17810791125

**Keywords:** prediction model, heart failure, hospital readmission, machine learning, cardiology, admissions, hospitalization

## Abstract

**Background:**

Patients with heart failure frequently face the possibility of rehospitalization following an initial hospital stay, placing a significant burden on both patients and health care systems. Accurate predictive tools are crucial for guiding clinical decision-making and optimizing patient care. However, the effectiveness of existing models tailored specifically to the Chinese population is still limited.

**Objective:**

This study aimed to formulate a predictive model for assessing the likelihood of readmission among patients diagnosed with heart failure.

**Methods:**

In this study, we analyzed data from 1948 patients with heart failure in a hospital in Sichuan Province between 2016 and 2019. By applying 3 variable selection strategies, 29 relevant variables were identified. Subsequently, we constructed 6 predictive models using different algorithms: logistic regression, support vector machine, gradient boosting machine, Extreme Gradient Boosting, multilayer perception, and graph convolutional networks.

**Results:**

The graph convolutional network model showed the highest prediction accuracy with an area under the receiver operating characteristic curve of 0.831, accuracy of 75%, sensitivity of 52.12%, and specificity of 90.25%.

**Conclusions:**

The model crafted in this study proves its effectiveness in forecasting the likelihood of readmission among patients with heart failure, thus serving as a crucial reference for clinical decision-making.

## Introduction

In contemporary society, with the advancement of civilization and the increasing aging population, cardiovascular diseases are emerging as a significant global health concern. Among these, heart failure (HF) stands out as a prevalent cardiovascular ailment characterized primarily by the heart’s inability to adequately fulfill the body’s blood supply requirements [1]. Despite notable strides in medical technology improving survival rates among patients with HF, the incidence of readmissions remains alarmingly high. Not only does readmission impose financial burdens on patients, but it also jeopardizes their quality of life and overall health status. Hence, predicting the likelihood of readmission in patients with HF is crucial for efficient medical management and care.

One of the primary motivations for this study is the significant challenge posed by the high readmission rates among patients with HF in China. These readmissions not only strain health care resources but also contribute to poorer patient outcomes, emphasizing the need for effective predictive models. Previous studies have demonstrated the potential of machine learning models in improving clinical decision-making, but their applicability to Chinese population with HF remains limited. This study aims to address this gap by developing a tailored predictive model that considers specific population characteristics and clinical contexts in China, contributing to more personalized and accurate interventions.

The process of readmission of a patient with HF is multifaceted, involving a plethora of intricate factors. Precisely identifying high-risk cohorts across various dimensions stands as a pivotal strategy in curtailing readmission rates [2]. By accurately forecasting this risk, health care teams can tailor patient-centric interventions while judiciously allocating health care resources. This necessitates a holistic approach encompassing not only the patient’s clinical status but also their lifestyle habits, psychological well-being, and socioeconomic circumstances, all of which may significantly influence their recovery trajectory and readmission susceptibility.

In the realm of medical research, both the logistic regression (LR) model and the Cox proportional hazards model serve as ubiquitous statistical analysis tools. However, their applicability is somewhat limited by inter-regional disparities, such as differences in health care infrastructure, patient demographics, and treatment practices across various geographic locations. These disparities can hinder the generalizability of models developed in one region when applied to another. Data truncation challenges further exacerbate challenges in model development, as clinical data sets often suffer from incomplete patient follow-up or missing records, particularly in real-world health care settings where longitudinal tracking can be difficult. With the rapid proliferation of big data services and technologies, coupled with substantial enhancements in parallel computing efficiency, the integration of machine learning in health care has experienced unprecedented growth. Machine learning–driven predictive models have demonstrated remarkable efficacy across various medical domains, including clinical diagnosis, disease prognosis, genetic analysis, and pharmacokinetics [3-6]. The success of these models owes to a diverse array of machine learning algorithms, encompassing linear regression, spectral clustering, hierarchical clustering, long short-term memory, gradient boosting trees, XGBoost, K-means clustering, and attention mechanisms. The versatility and formidable predictive power exhibited by these algorithms offer novel insights and tools for medical research.

In recent years, the application of machine learning in medical decision support systems has expanded significantly. For instance, Li et al [7] proposed a clinical decision system based on a deep belief network for patients with osteosarcoma, enhancing diagnostic accuracy and treatment efficiency, while Li et al [8] developed a predictive model for forecasting lymph node metastasis in patients with Ewing sarcoma, which is crucial for treatment planning and prognosis evaluation. Similarly, Li et al [8] introduced a machine learning–based predictive model for predicting lymph node metastasis in patients with Ewing sarcoma, and Dong et al [9] developed and validated a predictive model to evaluate the risk of bone metastasis in kidney cancer. These studies highlight the versatility of machine learning models across different cancer types and their utility in supporting clinical decision-making processes. Building on these advancements, this study explores the potential of the graph convolutional network (GCN) model in developing a readmission prediction model for patients with HF. By conducting a comparative analysis with conventional machine learning techniques, the study not only ensures data integrity but also seeks to provide an optimal strategy for predicting readmission of a patient with HF in China. This approach is vital in reducing the disease burden on patients and aligns with the broader goal of leveraging advanced machine learning to improve health care outcomes. Renowned as an efficient deep learning algorithm [10], the GCN model boasts formidable graph structure learning capabilities, adept at capturing intricate data relationships [11]. Concurrently, this study meticulously preserves data integrity during preprocessing to ensure the model’s predictive accuracy.

## Methods

### Source of Data

The data source for this study was the PhysioNet data portal [12]. The website provides a medical record–based database that integrates electronic medical records and external outcome data from admissions to a hospital in Sichuan Province, China, between 2016 and 2019 to create a retrospective HF database. The database encompasses a comprehensive array of 168 variables derived from 2008 patients with HF.

### Ethical Considerations

The Fourth People’s Hospital of Zigong’s Ethics Committee gave its approval for the creation of this database (approval number: 2020‐010) [13]. A separate publication [14] provides a full description of the database’s creation procedure. None of the data used in this study contained personal information.

### Target Population

**Figure 1.**
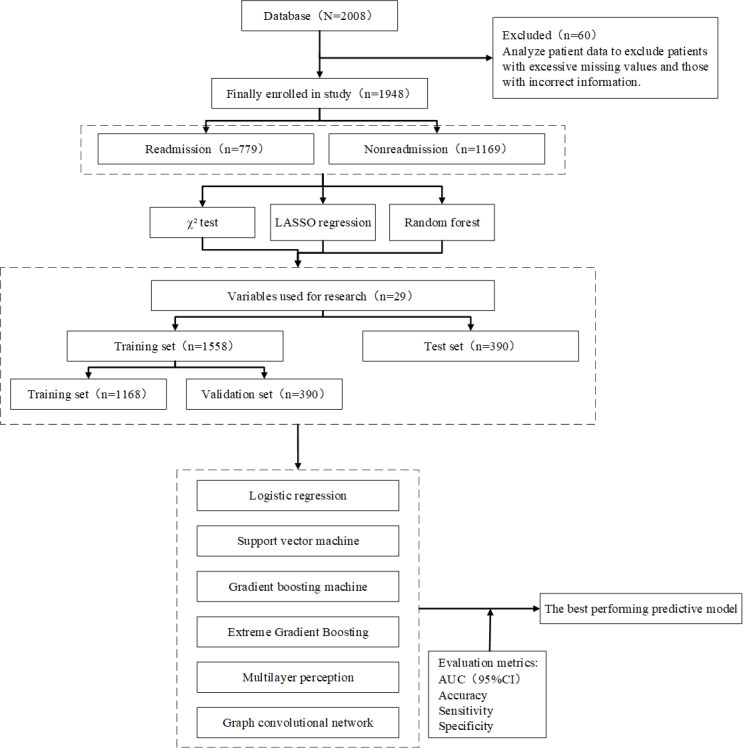
Research design. AUC: area under the receiver operating characteristic curve; LASSO: least absolute shrinkage and selection operator.

Following a comprehensive examination of the medical records of patients with HF, which encompassed a total of 2008 cases, the integrity of the data was meticulously evaluated in terms of its pertinence to patient outcomes. After a stringent procedure of data cleansing and scrutiny, the records of 1948 patients were conclusively deemed to satisfy the criteria of the study and were thus incorporated into this analysis, as illustrated in Figure 1.

### Data Preprocessing

Addressing data incompleteness emerged as a critical challenge during the course of this study. Specifically, characteristic variables exhibiting a missing rate of 50% or higher necessitated exclusion from the analysis due to the substantial extent of missing information. For other data with missing values, the k-nearest neighbor (KNN) algorithm [15] was deployed for imputation, with the K value set to 16 to optimize the imputation efficacy.

Upon completion of data imputation, information pertaining to the medications administered to patients during their hospitalization was reintegrated into the data set. Subsequently, a meticulous examination of the data ensued, whereby instances containing missing values were enumerated. Encouragingly, the incidence of such instances was minimal, facilitating their straightforward removal from the data set.

After a rigorous series of data preprocessing steps, 1948 study participants and 140 variables were included in the data set. Using these data, we define the target predictor variable as the probability that a patient will require hospital readmission for the specified condition within the next 6 months.

### Variable Selection

There were 140 major variables included in this analysis, including basic patient data such as quality-of life assessment, gender, vital signs, and socioeconomic factors, as well as the frequency of visits. Extensive documentation of baseline clinical characteristics was also conducted, encompassing blood markers (eg, hemoglobin levels, red blood cell counts, platelet counts), D-dimer levels, respiratory rate, blood pressure (both systolic and diastolic), and the Charlson Comorbidity Index. In addition, a number of distinct comorbidities, including diabetes, Alzheimer disease, and hepatopathy, were examined in the study.

To comprehensively identify variables associated with patient readmission outcomes at 6 months and ensure the inclusivity and representativeness of the predictive model’s variables, 3 distinct variable screening strategies were implemented within the training cohort. This multifaceted approach is designed to ensure the accuracy of the model’s construction and to broaden the applicability of its predictive capabilities through diverse methodological strategies, thereby enhancing its clinical utility. These strategies entailed (1) utilization of the *χ*^2^ test, which gauges the degree of association between the feature and the true label, thereby informing the selection process [16]. (2) Deployment of random forest, a technique generating multiple training sets by self-sampling the original data set and training a decision tree on each set. Subsequently, the ensemble method aggregates predictions through voting or averaging, enabling assessment of feature importance by evaluating their contributions to individual trees and comparing magnitudes across features [17]. (3) Adoption of least absolute shrinkage and selection operator (LASSO) regression, which achieves feature coefficient compression by introducing an L1 regularization term into the ordinary least squares method’s objective function. This regularization prompts some insignificant feature coefficients to converge to zero, effectively facilitating feature selection [18].

### Statistical Analysis

Every patient included in the research was subjected to a thorough statistical analysis. Categorical variables were statistically represented by frequencies and percentages, whereas continuous variables were expressed using means with SD and described with median and IQR. The “scikit-learn” Python package was used to implement the KNN method for estimation in order to handle missing values. In addition, the data set was randomly partitioned with an 8:2 sample size ratio into a test set and a training set. While the test set was used to validate the model, the training set was mainly used for variable screening and model construction.

A total of 6 different machine learning algorithms were used in this study to build the prediction model: XGBoost, LR, GCN, multilayer perception (MLP), support vector machine (SVM), and gradient boosting machine (GBM) [19-22]. The model’s performance was then thoroughly assessed in terms of accuracy, sensitivity, specificity, and area under the curve (AUC), which represents the area of the receiver operating characteristic (ROC) curve. The purpose of this evaluation was to determine how well the model predicted the likelihood of hospital readmission for patients with HF during the next 6 months. In addition, column-line plots were created to improve the interpretability of the model, making it easier for clinicians to understand the predictions of the model. The statistical analyses were carried out using Pycharm software and the Python programming language (version 3.10), using libraries like “scikit-learn [23],” “pandas [24],” “PyTorch [25],” and “PyTorch Geometric [26]”.

## Results

### Baseline Characteristics

This study encompassed 1948 participants and 140 variables, spanning various dimensions including baseline personal data, laboratory test results, medication use, and comorbidities. As depicted in Figure 1, out of the 1948 patients investigated, 779 patients experienced all-cause readmission, while 1169 patients did not. The data set was divided into two subsets: 390 patients formed the test set, and 1558 patients constituted the training set. Subsequently, the training set was further split into 2 subsets: 390 patients were allocated to the validation set, while 1168 patients remained in the training set.

### Variable Selection

**Figure 2.**
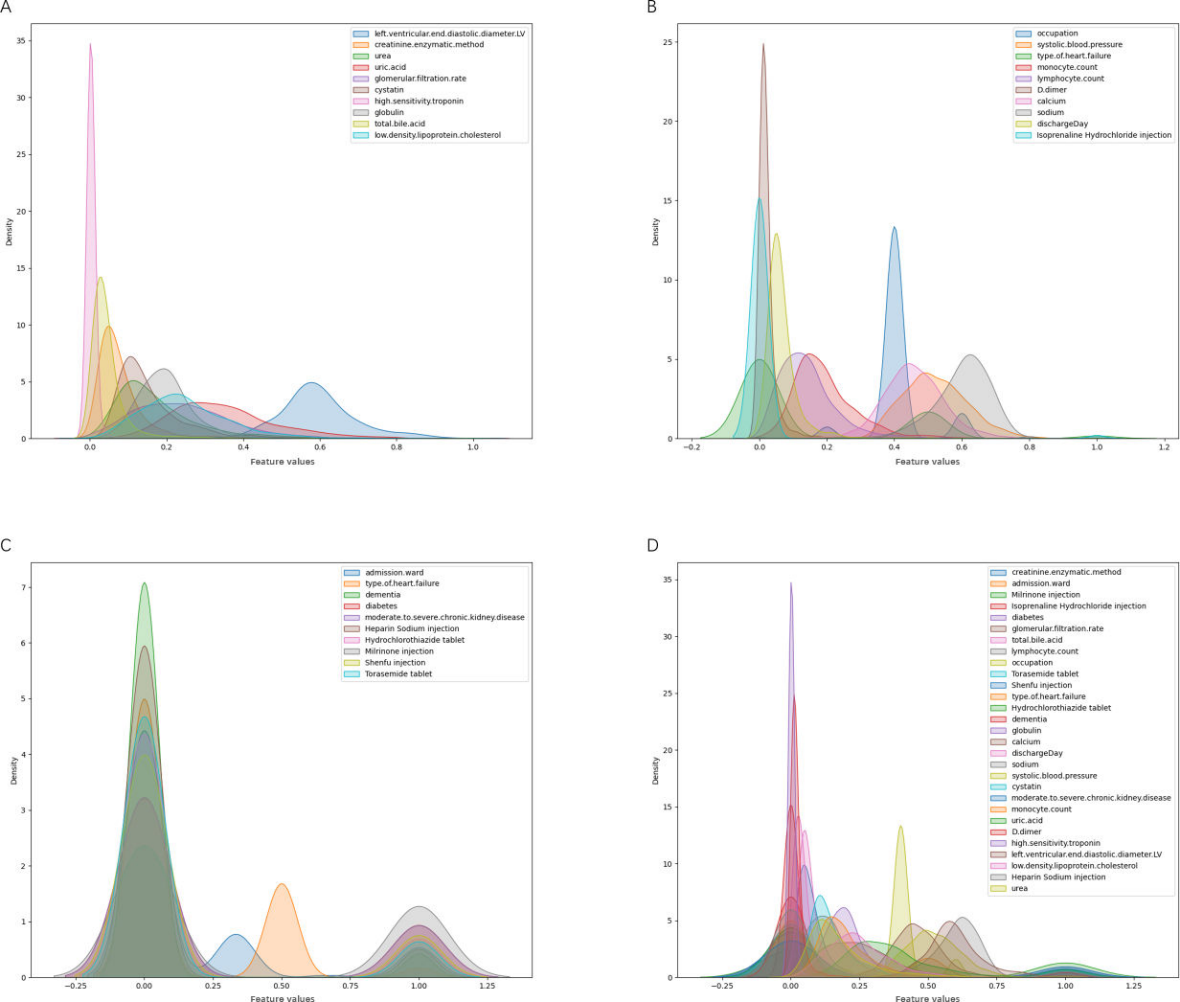
Kernel density maps for feature selection. (A) Random forest feature selection. (B) Least absolute shrinkage and selection operator feature selection. (C) *χ*^2^ feature selection. (D) Final selected features.

**Figure 3.**
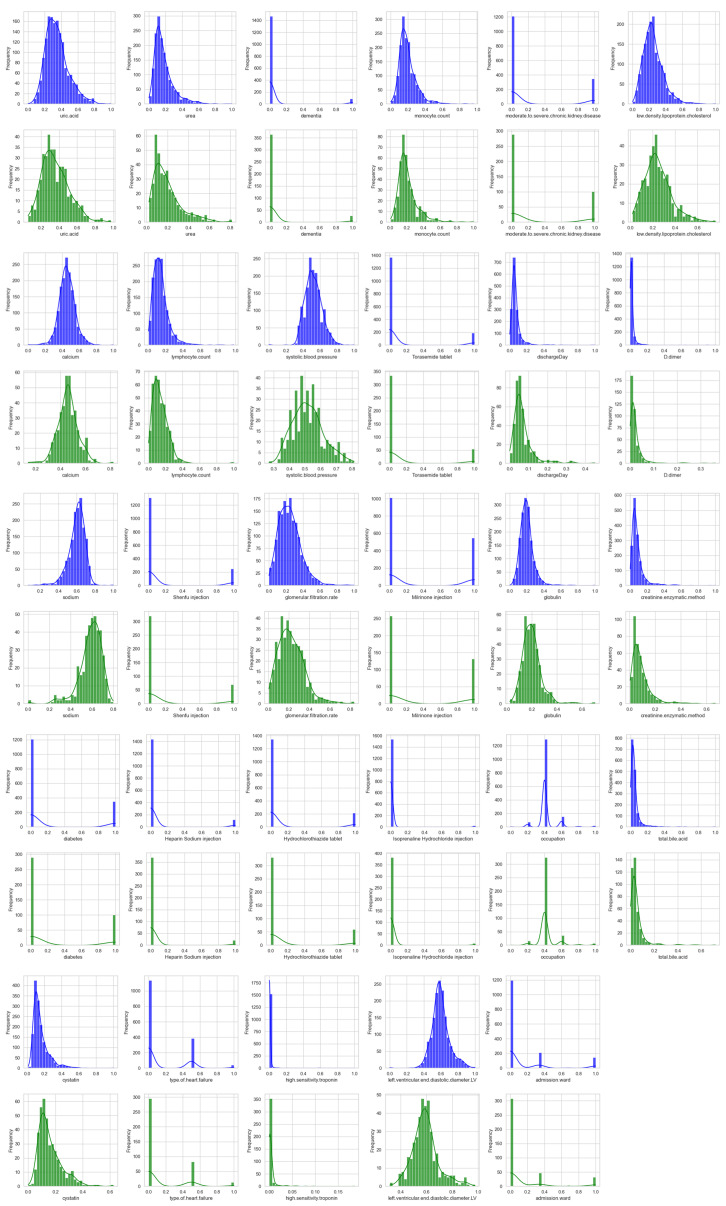
The specific distribution of the selected features in the training and test sets (the training set is represented in blue, and the test set is represented in green).

Figure 2 depicts the outcomes of the 3 screening methods and the final results. We have selected the union of these methods as our definitive feature set, which not only guarantees the completeness of the features but also enhances the model’s capacity to accommodate the intricacies of the data. In this study, most categorical and continuous variables exhibited significant differences between the nonreadmission group and the readmission group, indicating that these variables may play a crucial role in the risk of patient readmission. For example, variables such as diabetes, chronic kidney disease, type of HF, and the use of multiple medications showed highly significant statistical differences. However, some variables did not reach the conventional level of significance, such as isoproterenol injection, total bile acid, globulin, lymphocyte count, and monocyte count. Although these variables did not demonstrate statistical significance, after thorough discussions with experts in the relevant field, we believe that these variables may still hold clinical importance. Therefore, we decided to include them in the analysis to ensure the comprehensiveness and scientific rigor of the results. Ultimately, 29 feature variables were selected. Statistical descriptions in tabular form for both continuous and categorical variables are provided in Tables1  2, respectively. Furthermore, Figure 3 visually present the selected variables across the training set and test set.

**Table 1.** Composition ratios of categorical variables in selected features and their use.

Classification		Nonreadmitted (n=1169), n (%)	Readmitted (n=779), n (%)	*P* value
**Dementia**				.01
	No	1114 (95.3)	720 (92.43)	
	Yes	55 (4.7)	59 (7.57)	
**Diabetes**				<.001
	No	931 (79.64)	566 (72.66)	
	Yes	238 (20.36)	213 (27.34)	
**MSCKD^a^**				<.001
	No	938 (80.24)	560 (71.89)	
	Yes	231 (19.76)	219 (28.11)	
**Admission ward**				.01
	Cardiology	879 (75.19)	624 (80.10)	
	General ward	156 (13.34)	100 (12.84)	
	Intensive care unit	9 (0.77)	4 (0.51)	
	Others	125 (10.69)	51 (6.55)	
**Type of HF^b^**				<.001
	Both	800 (68.43)	632 (81.13)	
	Left	336 (28.74)	129 (16.56)	
	Right	33 (2.82)	18 (2.31)	
**Occupation**				<.001
	Officer	4 (0.34)	3 (0.39)	
	Worker	9 (0.77)	17 (2.18)	
	Urban resident	951 (81.35)	670 (86.01)	
	Farmer	142 (12.15)	48 (6.16)	
	Others	63 (5.39)	41 (5.27)	
**Heparin sodium injection**				<.001
	Nonuser	1059 (90.59)	747 (95.89)	
	User	110 (9.41)	32 (4.11)	
**Shenfu injection**				<.001
	Nonuser	1002 (85.71)	629 (80.74)	
	User	167 (14.29)	150 (19.26)	
**Hydrochlorothiazide tablet**				<.001
	Nonuser	983 (84.09)	693 (88.96)	
	User	186 (15.91)	86 (11.04)	
**Milrinone injection**				<.001
	Nonuser	802 (68.61)	467 (59.95)	
	User	367 (31.39)	312 (40.05)	
**Torasemide tablet**				<.001
	Nonuser	1048 (89.65)	657 (84.34)	
	User	121 (10.35)	122 (15.66)	
**Isoprenaline hydrochloride injection**				.07
	Nonuser	1147 (98.12)	773 (99.23)	
	User	22 (1.88)	6 (0.77)	

a MSCKD: moderate to severe chronic kidney disease.

b HF: heart failure.

**Table 2.** Statistical analysis of continuous variables in selected characteristics.

	Nonreadmitted (n=1169)		Readmitted (n=779)		*P* value
	Mean (SD)	Median (IQR)	Mean (SD)	Median (IQR)	
Urea	9.02 (5.26)	7.57 (5.7-10.7)	9.95 (5.17)	8.6 (6.24-12.2)	<.001
Calcium	2.29 (0.18)	2.28 (2.2-2.4)	2.31 (0.18)	2.3 (2.2-2.4)	<.001
SBP^a^	132.94 (24.46)	130.0 (118-150)	128.52 (24.44)	128.0 (110-143)	<.001
D-dimer	2.56 (5.81)	1.28 (0.8-2.2)	1.95 (3.58)	1.2 (0.71-2)	.03
Sodium	138.63 (4.74)	139.3 (136.5-141.8)	137.79 (4.97)	138.5 (135.15-141)	<.001
Uric acid	465.95 (160.52)	444 (353-554)	502.20 (172.4)	475.0 (377-604)	<.001
GFR^b^	72.51 (36.15)	70.44 (46.7-93.7)	64.70 (35.45)	58.01 (40.0-82.5)	<.001
Cystatin	1.76 (0.90)	1.48 (1.2-2.)	1.90 (0.92)	1.64 (1.27-2.29)	<.001
LVEDD^c^	51.91 (9.10)	51.0 (46-56.4)	53.37 (8.92)	52.31 (48.38-56.94)	<.001
LDL-C^d^	1.89 (0.72)	1.78 (1.4-2.3)	1.81 (0.73)	1.73 (1.32-2.14)	<.001
hs-Tn^e^	0.34 (2.40)	0.048 (0.02-0.12)	0.17 (0.67)	0.063 (0.03-0.12)	<.001
CRE^f^	102.11 (69.76)	82.2 (63-113)	113.72 (81.91)	94.07 (69.5-130.09)	<.001
Discharge day	8.69 (5.74)	7 (6-10)	10.66 (10.36)	8 (6-11)	<.001
Total bile acid	8.11 (10.72)	5.7 (3.3-8.9)	8.93 (13.32)	5.7 (3.2-9.3)	=.67
Globulin	28.65 (6.13)	27.9 (24.7-31.3)	28.32 (5.50)	28 (25-30.8)	.59
Lymphocyte count	1.03 (0.63)	0.94 (0.61-1.29)	1.04 (0.55)	0.94 (0.66-1.29)	.31
Monocyte count	0.48 (0.25)	0.42 (0.32-0.58)	0.47 (0.23)	0.42 (0.32-0.56)	.77

a SBP: systolic blood pressure.

b GFR: glomerular filtration rate.

c LVEDD: left ventricular end diastolic diameter.

d LDL-C: low-density lipoprotein cholesterol.

e hs-Tn: high sensitivity troponin.

f CRE: creatinine enzymatic method.

### Performance Evaluation Results of the Machine Learning Models

In this study, 3 independent strategies were used for variable screening, leading to the identification of 29 variables closely associated with the prognosis of patients with HF, which were subsequently included in the model construction process. A total of 6 models for predicting readmission risk have been developed with the aim of providing an accurate evaluation for patients with HF.

**Figure 4.**
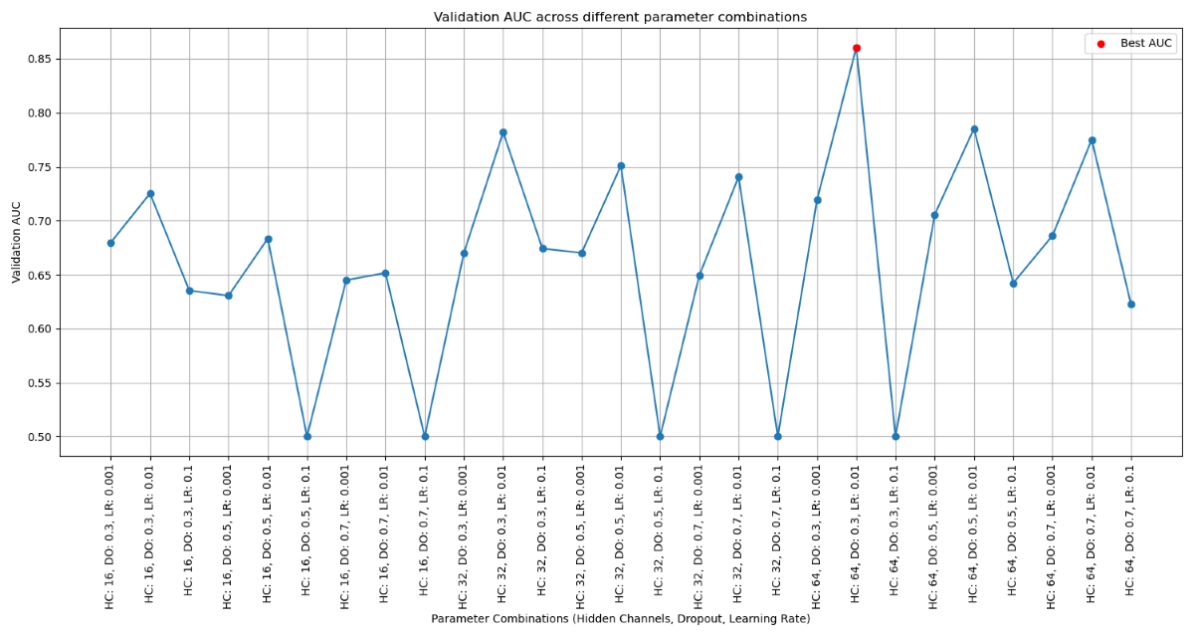
Graph convolutional network model hyperparameter tuning process. AUC: area under the receiver operating characteristic curve; DO: dropout; HC: hidden channels; LR: logistic regression.

During the construction of the GCN model, we systematically tuned the hyperparameters, including the number of neurons in the hidden layer, the dropout rate, and the learning rate, to optimize the model’s performance in predicting the risk of readmission in patients with recurrent HF. We evaluated the impact of various parameter combinations by calculating the AUC on the validation set and ultimately selected the combination that maximized the validation AUC. As shown in Figure 4, different parameter combinations led to significant variation in model performance, with some yielding higher AUC values and others leading to lower performance. In our experiment, we identified the configuration with the highest validation AUC, highlighted in red, which achieved the best performance. This systematic hyperparameter tuning process provided valuable insights into the model’s sensitivity, allowing us to select the settings with the highest potential for real-world application. Ultimately, we used this optimal parameter combination to construct our final model.

**Figure 5.**
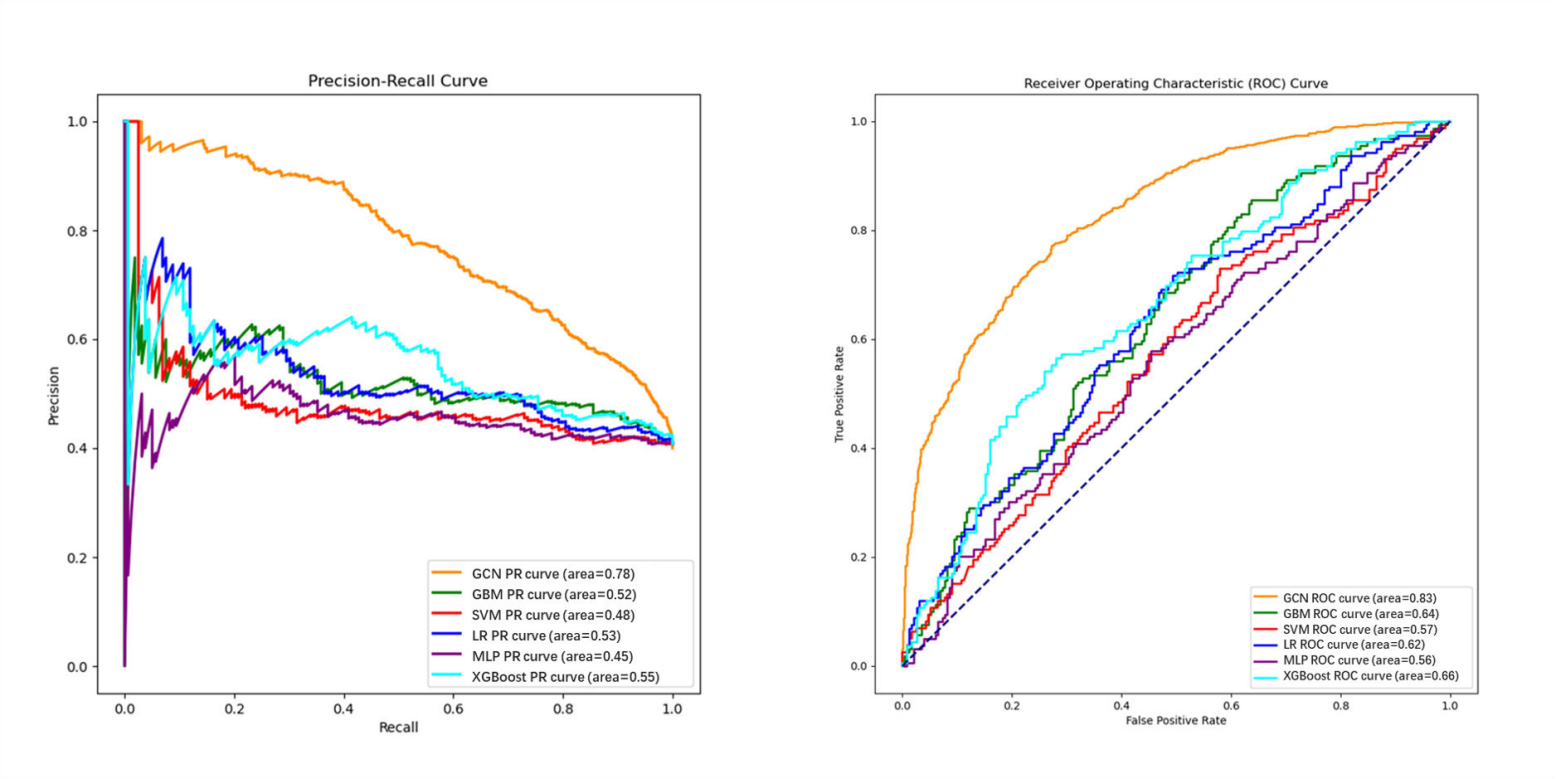
Performance comparison of different models. (A) Precision-recall (PR) curve. (B) Receiver operating characteristic (ROC) curve. GCN: graph convolutional network; GBM: gradient boosting machine; LR: logistic regression; MLP: multilayer perception; SVM: support vector machine; XGBoost: Extreme Gradient Boosting.

The outcomes of the 6 predictive models are presented in Table 3, while their respective performance comparisons are depicted in Figure 5. In the tabulated data, the GCN model distinguished itself with an AUC value of 0.831, significantly surpassing the other 5 models, whereas the MLP model exhibited the lowest AUC value of 0.564. Regarding prediction accuracy, the GCN, GBM, LR, and XGBoost models all achieved accuracies exceeding 60%, with the GCN model registering the highest accuracy of 75% and the MLP model yielding the lowest at 56.4%. In terms of sensitivity, the GCN, XGBoost, and MLP models demonstrated commendable performance, while in terms of specificity, the GCN, SVM, and XGBoost models outperformed others. Considering these 4 evaluation indices collectively, the GCN algorithm demonstrated the most effective prediction, followed by the XGBoost model.

**Table 3.** Predictive outcomes of heart failure readmissions for 6 machine learning algorithms.

Model	AUC^a^ (95% CI)	*P* value	Accuracy, %	Sensitivity, %	Specificity, %
LR^b^	0.624 (0.569-0.679)	<.001	0.613	31.45	81.82
SVM^c^	0.569 (0.510-0.628)	<.001	0.590	15.72	88.74
GBM^d^	0.637 (0.584-0.693)	<.001	0.610	33.33	80.09
XGBoost^e^	0.663 (0.608-0.718)	<.001	0.662	42.77	82.25
MLP^f^	0.564 (0.507-0.622)	<.001	0.564	40.88	67.10
GCN^g^	0.831 (0.813-0.849)	<.001	0.750	52.12	90.25

a AUC: area under the receiver operating characteristic curve.

b LR: logistic regression.

c SVM: support vector machine.

d GBM: gradient boosting machine.

e XGBoost: Extreme Gradient Boosting.

f MLP: multilayer perception.

g GCN: graph convolutional network.

### Crucial Predictors in Readmission Risk

**Figure 6.**
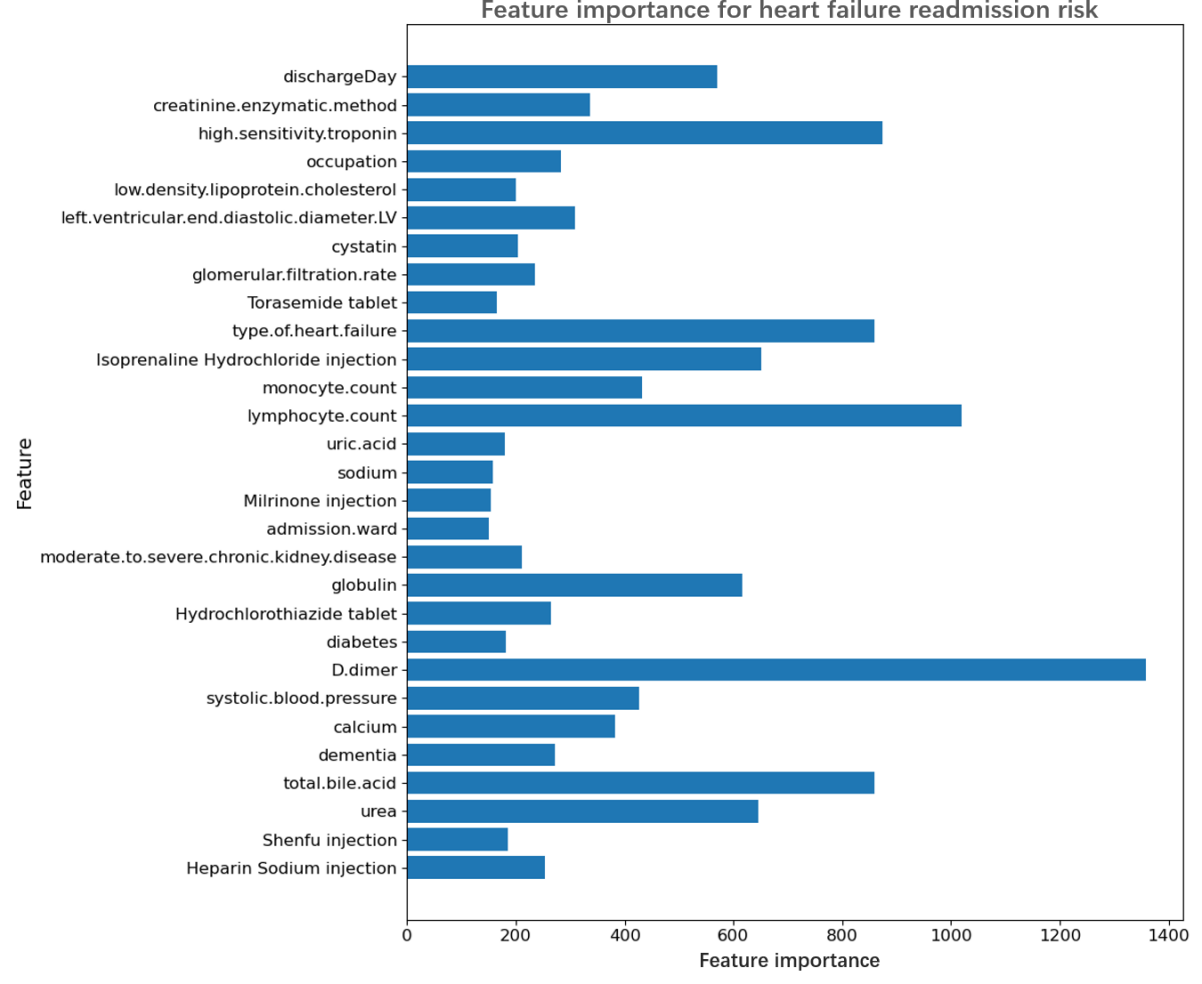
Importance of features for heart failure readmission risk.

**Figure 7.**
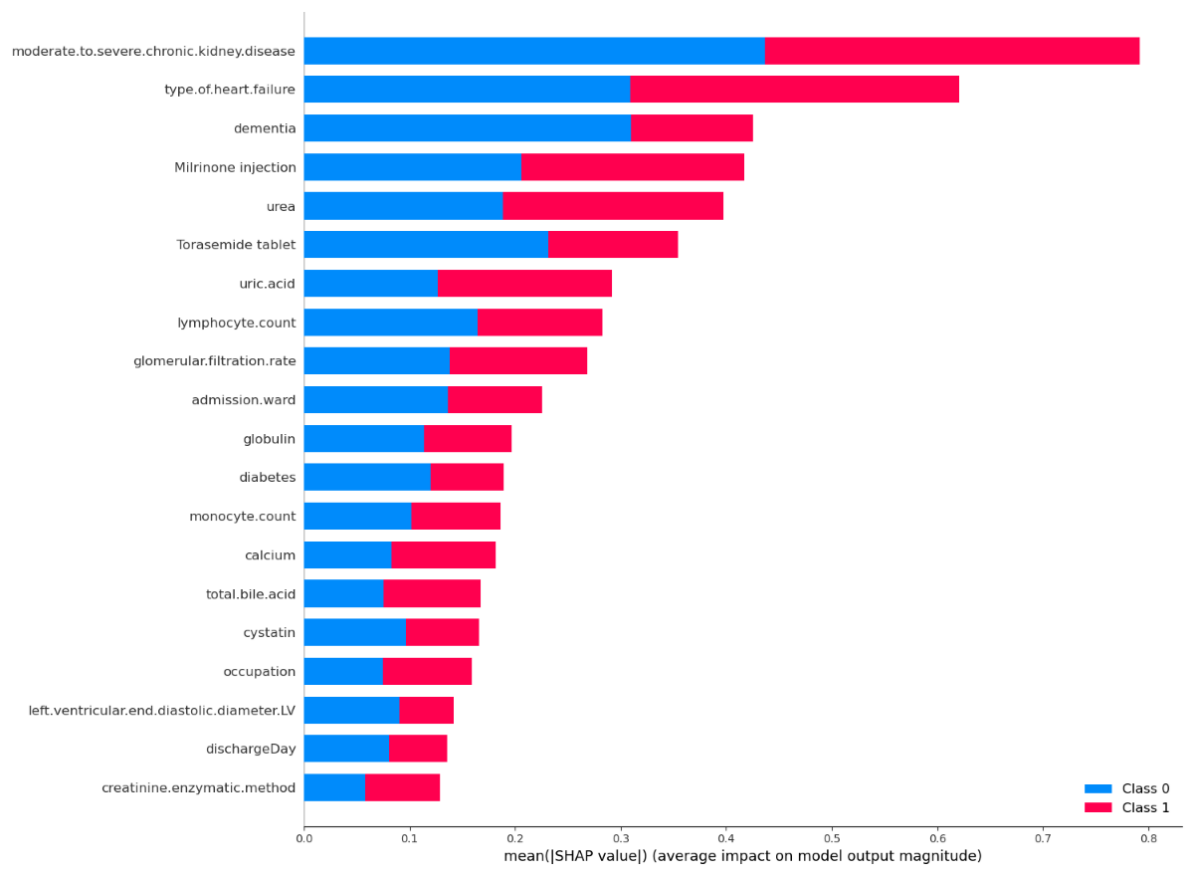
Shapley Additive Explanations (SHAP) value feature importance.

In this study, we used gradient attribution and SHAP (Shapley Additive Explanations) values to conduct an in-depth analysis of the model’s feature importance, aiming to gain a more comprehensive understanding of the model’s decision-making basis when predicting the risk of readmission in patients with HF. The gradient attribution method revealed the model’s sensitivity to changes in each feature (Figure 6). The results indicated that D-dimer and lymphocyte count are key features influencing the model’s output, with their importance scores significantly higher than those of other features. This suggests that the model is particularly sensitive to variations in these features when predicting readmission risk. In addition, features such as isoprenaline hydrochloride injection and high sensitivity troponin also showed high importance, implying that they may play a significant role in the clinical treatment of patients with HF, thereby substantially impacting the model’s predictive outcomes.The SHAP value analysis further revealed the specific contributions of each feature to the model’s predictions (Figure 7). SHAP values not only quantify feature importance but also illustrate the direction of these features’ influence on different prediction categories. The analysis showed that moderate to severe chronic kidney disease, type of HF, and dementia are the most influential features affecting the model’s prediction of readmission risk, with higher values of these features significantly increasing the likelihood of patient readmission. Furthermore, SHAP value analysis highlighted that certain features, such as uric acid and globulin, exhibit bidirectional influences in the model’s predictions, meaning that these features may have different effects on prediction outcomes depending on the context.

By integrating the results from both analysis methods, we reached the following conclusions: lymphocyte count emerged as a critical feature, displaying extremely high importance in gradient attribution and also showing a notable impact in SHAP value analysis, indicating its central role in predicting readmission risk for heart failure patients. Although features like D-dimer had high importance in gradient attribution, their impact was relatively smaller in SHAP value analysis, suggesting that these features might play a crucial role in the model’s local sensitivity. Conversely, features such as moderate to severe chronic kidney disease and type of HF were prominent in SHAP value analysis, underscoring their critical significance in the model’s overall predictions. By combining gradient attribution and SHAP value analysis, we not only identified the features crucial to the model’s predictions but also gained a more precise understanding of how these features function in different predictive contexts. This multidimensional analytical approach provides a solid foundation for formulating personalized treatment and management strategies and contributes to improving the accuracy of readmission risk prediction for patients.

## Discussion

Clinical prognosis of patients with HF is highly variable and closely related to the course and severity of the illness. Building a predictive model for 6-month readmission risk that is specifically tailored to the Chinese population is crucial for both patients and health care professionals in the global context of precision prevention and treatment strategies. Clinical and biochemical indicators are important points of reference for physicians because of their accessibility and ease of interpretation, even though a variety of complex factors influence the readmission risk of patients with HF. In this study, alongside integrating clinical and biochemical indicators, considerations were extended to encompass patients’ state of consciousness, verbal responses, mobility, and socioeconomic factors such as substance use and occupational status [27].

The study was conducted with the objective of accurately forecasting the 6-month readmission risk for patients with HF. To this end, 6 predictive models were developed and validated, using 3 distinct variable screening methods that incorporated 29 characteristic variables. The models’ performance was rigorously assessed using 4 key metrics: AUC, accuracy, sensitivity, and specificity. Among all the models tested, the GCN-based model emerged as the most effective, as evidenced by its outstanding AUC of 0.866, an accuracy rate of 0.75%, a sensitivity of 52.12%, and a specificity of 90.25%. These results underscore its superior predictive prowess.

By elucidating optimal readmission strategies for patients with HF in China, this study aims to provide robust support for clinical decision-making, enabling physicians to conduct more precise assessments of readmission risk. Building on this foundation, it seeks to alleviate the disease burden and improve the quality of life for patients with HF. This study innovatively applies the GCN model to predict readmission risk for patients with HF, leveraging its unique graph structure learning capability to effectively capture and use complex structural relationships within the data, demonstrating significant advantages in handling high-dimensional, heterogeneous medical data. Through rigorous data preprocessing and variable selection strategies, this study ensures the scientific validity and reliability of the model’s predictions, enhancing the credibility of the findings. The systematic comparative analysis comprehensively evaluates the performance of GCN against various traditional machine learning models, with results showing GCN’s superior performance across key metrics, further confirming its effectiveness in predicting HF readmission risk. This research not only expands the application boundaries of GCN in medical prediction but also provides precise tools and methods for predicting readmission risk in Chinese patients with HF, holding significant clinical value for reducing readmission rates, alleviating healthcare burdens, and improving patient quality of life.

In this study, we systematically compared various machine learning models, including XGBoost, LR, GCN, MLP, SVM, and GBM. Although these models demonstrated certain predictive capabilities across different tasks, GCN exhibited significant advantages, especially in tasks involving complex structured data. Unlike other traditional models, GCN can effectively use the latent complex structures within the data, aggregating relevant information to generate richer and more representative feature representations. In contrast, while XGBoost and GBM excel at handling high-dimensional features, they cannot directly capture the structural relationships within the data, leading to limited performance when dealing with complex structured data. Similarly, MLP and SVM, as typical nonstructured models, are unable to leverage relational information within the data, relying solely on independent features for prediction, which weakens their ability to capture complex dependencies among features. Although LR has the advantage of being simple and easy to use, its linear nature makes it difficult to handle complex nonlinear relationships. GCN, through its unique convolutional operations, not only effectively integrates both local and global information but also maintains stable performance in sparse data environments. This ability to aggregate information and process complex structured data enabled GCN to significantly outperform other models in predicting the risk of rehospitalization among patients with HF. Therefore, we chose GCN as our primary model to fully exploit the complex structural information within the data and enhance prediction accuracy.

The international medical community considers research on predicting the risk of readmission in patients with HF to be extremely important [28-30]. Research has repeatedly shown that the use of GCN results in better predictive performance when compared to other algorithms. These findings were derived from a predictive model encompassing 1948 patients and 29 associated factors, showcasing excellent performance relative to prior studies [28,31-34].

However, it’s important to recognize that the comparison between different predictive models isn’t always straightforward, given the variance in methodologies and variables used. Currently, most machine learning–based HF readmission prediction models lack validation in a prospective cohort of Chinese patients with HF. Notably, significant disparities exist between Western and Chinese populations concerning dietary habits, ethnic composition, and disease prevalence [35,36]. Consequently, caution should be exercised when extrapolating models constructed based on Western populations to Chinese or other Asian populations, to avoid potential misinterpretations and contentious conclusions.

Although this study’s results indicate that readmission risk of patients with HF can be accurately predicted, it should be noted that there are a number of limitations. First, while this study used 29 characteristic variables to develop a predictive model, the practical clinical application of using such a large number of variables is constrained. In a real-world clinical setting, obtaining all the feature variables used in this study may not always be feasible due to challenges in data collection, especially in resource-limited environments. This represents a significant limitation, as the practical utility of the model in daily clinical practice could be reduced by the availability of these variables. Future work should aim to simplify the model by identifying the most critical predictive features, thus improving its clinical applicability without compromising accuracy.

First, it was impossible to establish a clear causal link between the chosen factors and patient readmission due to the retrospective analysis of databases. Nonetheless, the study mitigated this limitation by using diverse screening and modeling strategies, coupled with result validation, to bolster the robustness of its findings and provide a strong quantitative basis for future prospective studies.

Second, while multicenter studies are typically deemed more representative, it is worth noting that single-center predictive models have yielded significant results in clinical research. This study, based on single-center data, holds clinical significance and reinforces the value demonstrated by single-center studies in advancing our understanding of predictive modeling in healthcare.

Third, the absence of ECG-derived feature information in the database limited the consideration of potential predictors in this study. However, the study thoroughly explored the predictive role of available variables in predicting 6-month readmission risk. The data set for this study was derived from a database of Chinese patients with HF, which may have specific applicability to Asian populations. Nonetheless, constructing more generalizable models will necessitate comprehensive databases encompassing a broader spectrum of populations.

Furthermore, akin to many prior studies, this study did not extensively analyze the specific causes of readmission. Nonetheless, considering that HF predominantly affects the elderly population, curtailing all-cause readmission rates is paramount to enhancing patient survival quality. This study’s model demonstrated excellent specificity and overall accuracy, suggesting that it is a highly effective tool for identifying low-risk individuals. Furthermore, the model was able to predict that most patients would not require readmission to the hospital within 6 months of their discharge. This insight is crucial for judiciously allocating health care resources and channeling additional resources toward high-risk individuals, thereby diminishing readmission rates and enhancing their quality of survival.

Finally, this study methodically selected and compared the 6 most prevalent methods among various traditional statistical models and machine learning modeling techniques. The findings underscored the robustness and rationality of the modeling methods used in this study, underscoring their suitability for predictive modeling in the context of HF readmission.

In future work, we plan to further expand and diversify our data set to enhance the model’s generalization ability across different patient populations and explore extending the data set to a broader range of geographic regions and hospital systems. We will focus on optimizing the GCN model and exploring the integration of other advanced machine learning models, such as deep neural networks and ensemble learning methods, to improve the accuracy and robustness of HF readmission risk prediction.

In addition, we intend to further develop more transparent model interpretation frameworks, enabling clinicians to better understand and trust the model’s outputs. We also plan to strengthen our collaboration with clinicians and medical experts to ensure the model’s effectiveness in real-world clinical settings. To further facilitate practical application, we aim to develop a web-based calculator that allows healthcare providers to easily input patient data and quickly obtain predictions of readmission risk. This tool will enhance the accessibility and usability of the model in clinical practice. Furthermore, we plan to apply our research findings to the risk prediction of other chronic diseases, thereby expanding its potential applications in medical decision support.

Finally, we will closely monitor the ethical considerations associated with the model’s real-world application, evaluating its potential impact on patients and health care systems to ensure that our research remains safe and socially acceptable in clinical practice.

In conclusion, this study successfully developed a predictive model using a GCN with 29 variables to forecast the risk of readmission for patients with HF over a 6-month period. Through comprehensive assessment using 4 key evaluation metrics, the model exhibited exceptional performance and yielded convincing validation results. This important discovery provides a critical point of reference for therapeutic treatment and diagnostic judgment with regard to Chinese individuals suffering from HF. Furthermore, it provides insightful information for the next research on HF readmission in Asian populations.

## Abbreviations

AUC: area under the receiver operating characteristic curve
GBM: gradient boosting machine
GCN: graph convolutional network
HF: heart failure
KNN: k-nearest neighbor
LASSO: least absolute shrinkage and selection operator
LR: logistic regression
MLP: multilayer perception
SHAP: Shapley Additive Explanations
SVM: support vector machine
XGBoost: Extreme Gradient Boosting
